# Short‐Term Effects of Attaching Animal‐Borne Devices on the Behavior of Juvenile Green Turtles

**DOI:** 10.1002/ece3.70707

**Published:** 2024-12-23

**Authors:** Nathan J. Robinson, Ruth Doñate‐Ordóñez, Damianos Chatzievangelou, Annabelle M. L. Brooks, Jack Cuffley, Candace Y. A. Fields, Sebastian Hoefer, Theodora Pinou, Alexander Smith, Sophie Mills

**Affiliations:** ^1^ Institut de Ciències del Mar Spanish National Research Council (CSIC) Barcelona Spain; ^2^ Fundación Oceanogràfic Ciudad de Las Artes y Las Ciencias Valencia Spain; ^3^ University of Algarve Faro Portugal; ^4^ Cape Eleuthera Institute Cape Eleuthera Island School Rock Sound The Bahamas; ^5^ Institute of Environment, Department of Biological Sciences Florida International University North Miami Florida USA; ^6^ College of Science and Engineering James Cook University Townsville Queensland Australia; ^7^ Biology Department Western Connecticut State University Danbury Connecticut USA; ^8^ School of Biological Sciences Monash University Clayton Victoria Australia

**Keywords:** animal‐borne cameras, *Chelonia mydas*, handling stress, sea turtles, telemetry, unoccupied aerial vehicles

## Abstract

The use of animal‐borne devices (= biologgers) has revolutionized the study of marine megafauna, yet there remains a paucity of data concerning the behavioral and physiological impacts of biologger attachment and retention. Here, we used animal‐borne cameras to characterize the behavior and dive duration of juvenile green turtles ( *Chelonia mydas* ) in The Bahamas for up to 210 min after biologger deployment (*n* = 58). For a “control,” we used unoccupied aerial vehicles (UAVs) to collect comparable data from nonhandled green turtles (*n* = 25) in the same habitats. Animal‐borne footage revealed that immediately after release turtles spent 70%–80% of their time swimming with a mean dive duration of 45.3 ± 34.3 s (SD). Over time, the percentage of time spent swimming decreased alongside an increase in dive duration until reaching a plateau around 90 min. However, the “control” UAV data for time spent swimming and dive durations were more comparable to the behaviors observed immediately after biologger deployment than during the plateau. We observed no significant differences in dive durations based on body size, and differences in behaviors based on body size were also minimal. We conclude that the effects of handling stress and biologger attachment on the behavior and dive duration of juvenile green turtles are evident up to 90 min postdeployment. After that, it is possible that either: (1) the effects of biologger deployment and retention are negligible, but UAVs may produce biased data that overestimates the proportion of time turtles typically spend swimming or (2) longer durations (> 210 min) are necessary for turtle behaviors to return to nonhandled levels and UAVs accurately represent the proportion of time turtles typically spend swimming. Answering this question, alongside further research into the physiological and behavioral implications of handling stress and biologger attachment, is essential to improve ethical biologging guidelines for sea turtles.

## Graphical abstract

The increasing use of animal‐borne devices (= biologgers) has revolutionized the study of marine megafauna, yet there remains a distinct paucity of data concerning the behavioral and physiological impacts of handling stress and biologger attachment. Here, we assessed short‐term (< 210 min) trends in the behavior and diving rate of juvenile green turtles ( *Chelonia mydas* ) in The Bahamas immediately after capture and biologger deployment.

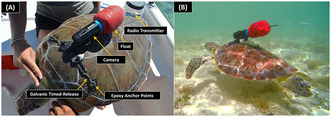

## 1 Introduction

Animal‐borne devices (hereafter referred to as biologgers and includes biotelemetry devices) have revolutionized our understanding of the behavior, physiology, and ecology of many marine megafauna (Wilmers et al. 2015; Watanabe and Papastamatiou 2023). With the capacity to record continual streams of data from free‐ranging animals, biologgers provide a practical method for studying species that are challenging to track via other means (Hays et al. 2016). Yet, as the use of biologgers has grown increasingly widespread (McIntyre 2014; Robinson et al. 2023), so have calls for empirical studies on how the attachment and retention of these devices affect the tagged animals (Wilson et al. 2018; Williams et al. 2020). With implications for ethics, animal welfare, and conservation management (McMahon, Hindell, and Harcourt 2012), such studies may be particularly urgent for endangered species such as sea turtles.

Deploying biologgers onto megafauna inherently requires some physical interaction with the study organism. To capture sea turtles in water, the most common methods are to use either a dip‐net to scoop up turtles from a boat or snorkelers to capture turtles by hand (Ehrhart and Ogren 1999). Turtles are then typically held out of the water while routine data are collected (e.g., ID tags checked/deployed, morphometric measurements) and, if needed, biologging devices are attached. In some instances, biologging devices are attached by drilling fixture holes through the marginal rim of the carapace (e.g., Hill et al. 2017) or with suction cups (e.g., Hounslow et al. 2021), but most devices are attached to the carapace using epoxy (Coyne et al. 2008; Mansfield et al. 2012; Hart, Guzy, and Smith 2021). Epoxy requires time to set (between 5 min and several hours depending on the product) and has an exothermic reaction (Sypniewski et al. 2016). Once the epoxy has set, the turtles are generally released. Although it should be ensured that animals are always handled in the most stress‐free manner, it should not be ignored that this process still likely causes a stress–response in the animal and may influence their behavior postrelease. Indeed, studies have revealed that several hours after capture, sea turtles have elevated levels of corticosterone (Gregory et al. 1996)—a typical marker for stress in wild animals (Baker, Gobush, and Vynne 2013).

Beyond handling stress, the retention of a biologging device could also alter a turtle's behavior. It is difficult to determine if an animal is conscious of an attached biologging device, but sea turtles are known to routinely scratch their shells on objects presumably for self‐cleaning (Harvey‐Carroll et al. 2024). Thus, if a turtle senses the presence of a biologger, it may spend more time scratching its shell. Biologgers could also influence a turtle's movement by affecting its buoyancy or hydrodynamic profile. Buoyancy issues are generally minimized by ensuring the device is as neutrally buoyant as possible, but it is more challenging to reduce hydrodynamic drag. In fact, even low‐profile satellite transmitters may increase drag by up to 30% when deployed on turtles with straight carapace lengths < 50 cm (Jones et al. 2013). Greater hydrodynamic drag would increase the energetic cost of swimming while simultaneously reducing the animal's capacity to evade predators or capture prey.

It can be relatively challenging to accurately determine how an animal's behavior is affected by handling stress and biologger attachment as it requires generating comparable behavioral data from individuals that have not been handled or carrying biologgers. Unoccupied aerial vehicles (UAVs) can provide practical tools to collect such data as they can track and follow the movements of large marine animals in shallow‐water habitats (e.g., Robinson et al. 2020). Moreover, sea turtles appear largely unresponsive to UAVs flown above 10 m altitude (Bevan et al. 2018). When behavior data from nontagged individuals are not available, another method to assess the effect of deploying a biologging device is to assume that after a given length of time an animal with a biologger attached will return to a “normal” baseline behavioral pattern. Indeed, some studies have compared the behavioral patterns of animals immediately postdeployment to those several hours to days later (e.g., Thomson and Heithaus 2014; Kline, Ripperger, and Carter 2021; LaRochelle et al. 2022).

Only a single study, Thomson and Heithaus (2014), has previously attempted to directly assess how handling stress related to biologger attachment influences green turtle ( *Chelonia mydas* ) behavior. This study, which focused on subadult and adult green turtles in Australia, used animal‐borne cameras and revealed that turtles exhibited “excessive” swimming immediately after deployment relative to those filmed after a 24‐h delay. Yet it is not known if behavioral changes are also observed over shorter time scales nor whether all life stages of green turtles exhibit similar responses. For example, it has also been shown that body size differences in sea turtles appear to be a key factor influencing their “startle” response to nearby snorkelers (Siegfried et al. 2023) and that posthandling stress hormone levels in larger turtles tend to drop faster than in smaller turtles (Gregory et al. 1996; Jessop and Hamann 2005). It may therefore be feasible to expect that larger turtles would return to normal behaviors postrelease sooner than smaller turtles.

Here, we assessed the short‐term impacts (up to 3.5 h) of biologger attachment and retention on the behavior of juvenile green turtles in Eleuthera, The Bahamas, by using a combination of animal‐borne cameras and UAVs. We also assessed whether the behavioral response differs between turtles of different size classes. As such, we had three specific objectives: (1) to describe the behavior of juvenile green turtles in the hours immediately following capture and biologger deployment, (2) to compare the behavior of turtles with biologging devices to the behavior from nonhandled animals that were recorded via UAV, and (3) to determine whether behavioral change postbiologger deployment differed between turtles based on size.

## 2 Methodology

### 2.1 Study Site

**FIGURE 1:**
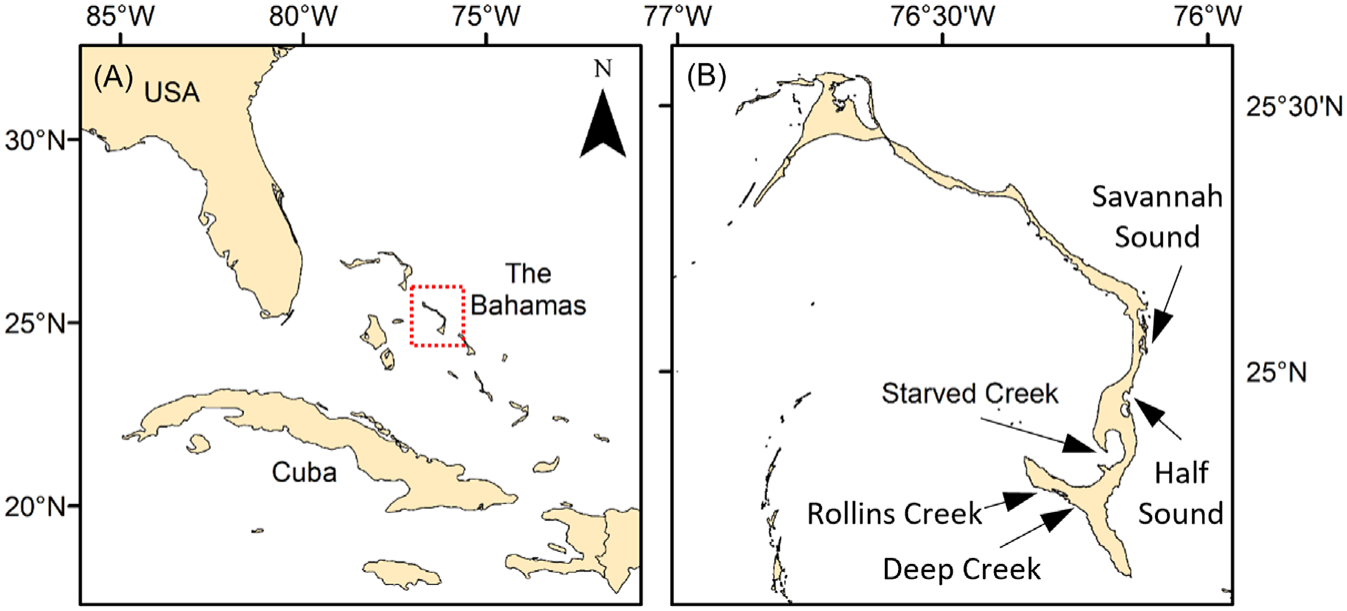
(Left) Map indicating the location of Eleuthera (red square) in The Bahamas. (Right) Map of Eleuthera indicating the five sites where turtles were sampled.

We deployed animal‐borne cameras on juvenile green turtles caught on the southern end of the island of Eleuthera (25° 06′ 00″ N, 76° 07′ 59″ W; Figure 1), The Bahamas. Specifically, we sampled turtles found in five mangrove creeks: Rollins Creek, Deep Creek, Starved Creek, Savannah Sound, and Half Sound. The creeks were shallow (typically < 2 m depth and up to a maximum of 5 m depth), with a mix of sandy, muddy, and rocky substrates. Each creek is a known foraging area for green turtles and beds of seagrass ( *Thalassia testudinum* ) and various algal species were found at all locations.

### 2.2 TurtleCam Deployment

**FIGURE 2:**
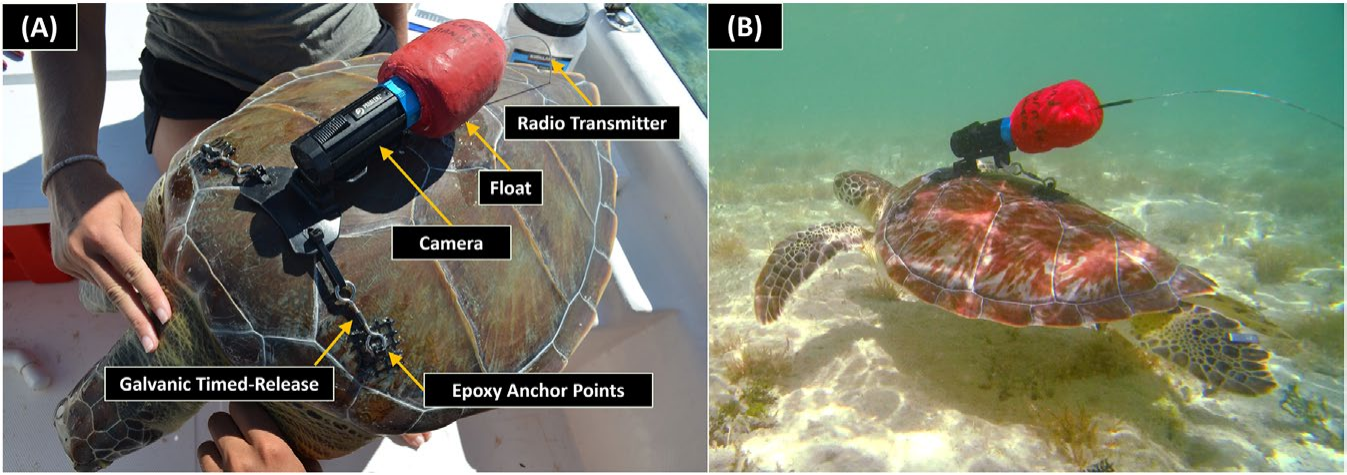
(A) Image of the TurtleCam and the mechanism for temporarily attaching it to a sea turtle's carapace. (B) A free‐swimming green turtle with a TurtleCam attached.

Between September 2018 and March 2020, we captured juvenile green turtles using a modified rodeo technique as described in Ehrhart and Ogren (1999). In short, all turtles were pursued by boat after sighting. When approaching within a few meters of the animal, a snorkeler would enter the water and restrain the turtle at the base of the front flippers. If unsuccessful, additional snorkelers would enter the water until the turtle was caught and brought onboard the boat. Caught turtles were examined for identification tags. If none were found, we attached individually numbered metal inconel tags on the trailing edge of the front flippers. We also took photos for photo identification (Mills et al. 2023) and measured straight carapace length (SCL) and width (SCW) using calipers. Animals were then visually assessed for external injuries or abnormalities. Turtles with no visible injuries or abnormalities and > 30 cm SCL were selected for the deployment of an animal‐borne camera (Figure 2). We only selected animals > 30 cm SCL based on the size of the animal‐borne camera relative to the turtle's carapace.

The animal‐borne cameras, hereafter referred to as TurtleCams, were custom built by attaching a VHF radio transmitter (MOD‐050‐2, Lotek, USA) and a section of buoyant foam to a dive camera (DiveCamera+, Paralenz, Denmark). Each TurtleCam was 18‐cm long (excluding the flexible antennae), was 7 cm at its widest point, and had less than 100 g of buoyancy in water. Each TurtleCam had in‐built sensors that measured temperature (±0.1°C) and depth (±0.1 m) every second. To deploy the TurtleCam, we used 5‐min epoxy (KwikWeld, USA) to affix three pieces of 4 × 4 cm plastic mesh. Two pieces of the mesh were placed bilaterally toward the front of the animal's carapace, while the third was placed along the midline toward the back of the carapace. We attached galvanic timed releases (AA2, International Fishing Limited, New Zealand) to each piece of plastic mesh using zip ties. Finally, additional zip ties were used to connect the galvanic timed releases to the TurtleCam. It took between 15 and 30 min to attach a TurtleCam and collect all the tagging and morphometric data. After the TurtleCam was successfully attached and video recording initiated, we released the turtle within 100 m of its original encounter location. The galvanic timed releases corroded within a predetermined length of time (3–4 h), releasing the TurtleCam and allowing it to float to the surface. We then used a unidirectional antenna and VHF radio receiver to track and recover the TurtleCam. Once the TurtleCam was recovered, we downloaded all the recorded footage.

Prior to the detachment of the TurtleCam from the turtle's carapace, we refrained from approaching within 1 km of the turtle's release location to mitigate any further effects on the behavior of the turtle with the biologging devices. In addition, we never saw any other vessels in our study area on any sampling days. All animals were caught between 10:00 and 14:00 to minimize any confounding effects associated with diel variation in green turtle behavior (e.g., Hart et al. 2016) on our analyses.

### 2.3 UAV Methodology

We used a DJI Mavic 2 Pro to conduct haphazard surveys of the same creek systems as surveyed for TurtleCams. Initially, the drone was flown at an altitude of 30 m until a turtle was spotted. At this point, we positioned the camera perpendicular to the surface of the water and lowered the drone to an altitude of 15 m. We then began recording, ensuring that the turtle remained within the center of the frame for as long as possible based on battery life. This was repeated up to three times per day per creek and each survey was conducted in a separate area of the creek to ensure that the same turtle was not recorded multiple times. UAV surveys were conducted on different days to those when turtles were being sampled for TurtleCams to ensure that any individuals filmed via the UAVs had not been handled by us in the past 24 h. UAV surveys were always conducted between 10:00 and 17:00 to match with data collected via the TurtleCam (the latest a TurtleCam would be deployed was 14:00 and it could potentially record for 3 h).

### 2.4 Video Analysis

We annotated all footage (per second) recorded by the TurtleCam and the UAV based on the following behaviors: swimming, surfacing (including breathing), resting, feeding, social interactions, or other (for a full description of each behavior see Table 1 and for examples see the embedded Video 1). All videos were analyzed by a single individual to avoid errors associated with subjective variation in behavioral assignments. Furthermore, a single 10‐min section was randomly selected from each turtle and annotated for a second time to double‐check accuracy. If > 5% of the behavioral labels were not consistent between the original labels, the entire 3 h video was relabeled. We also quantified dive durations by calculating the time between each surface event and then calculated the mean water temperature using the TurtleCam's in‐built temperature sensor. For the TurtleCam footage, we divided the annotated dataset into 30‐min segments starting from when the turtle was returned to the water so that we could determine how animals' behaviors and dive durations changed over time. In contrast, the UAV footage was considered a single time unit as we were not interested in changes over time. We only used videos from TurtleCams and UAVs that generated over 2 h or 10 min of usable footage, respectively.

**TABLE 1:** Ethogram describing the different behavioral categories that were observed from both the TurtleCam and the UAV footage.

Behavior	Description
Swimming	The turtle swims using its front flippers and without touching the seafloor or the water's surface
Surfacing	We considered that surfacing began the instant that the animal's head breaks the surface of the water and continued until the turtle's head was resubmerged and, importantly, it began to actively swim away from the surface. As such, a continually labeled surfacing event could include several individual breaths if the animal remained at the surface between breaths
Resting	When the turtle was inactive (e.g., not swimming, breathing, feeding, socializing, crawling, or digging). This included when turtles rested both in the open or under substrate (e.g., overhanging rocks or coral) but did not include when resting at the surface between breaths (as this would be considered surfacing). It should be noted that due to the wave action at the study site, it was impossible to discern whether small back‐and‐forth movements conducted by turtles when resting under shelter where passive or representing an active attempt to scratch the carapace
Feeding	Turtle actively feeding. This was often inferred via the movements of the turtle's head and accompanied by swallowing movement of the neck as the angle of both the TurtleCam and the UAV did not allow for a clear view of the turtle's beak
Socializing	When the turtle of interest was following, approaching, circling, biting, or interacting with any other turtle
Other	Any behavior that was not categorized earlier. Typically, this was either digging or crawling

**VIDEO 1:** Video examples of each of the catagorized behaviors recorded via the TurtleCam. Video content can be viewed at https://onlinelibrary.wiley.com/doi/10.1002/ece3.70707

Turtles were separated into those with SCLs ≤ 50 cm and those with SCLs > 50 cm. This distinction was chosen as it was the mid‐point between the minimum size (SCL: 30 cm) chosen to deploy a TurtleCam and the maximum size (SCL: ~70 cm) for green turtles at the study site (Siegfried et al. 2021) and close to the mean measured size of our samples (see “Results” next). In addition, this arbitrary division also helped to ensure relatively even sample sizes between each size grouping. For the turtles with TurtleCams, SCL was determined via measurements taken by hand while the turtles were on the boat. For the UAV footage, we measured the length in pixels of a 50‐cm measuring stick at ground level when recorded by the UAV's camera at an altitude of 15 m and then used this as a scale to measure the length in pixels of the turtle's carapace when at the surface. We accept that there is likely at least 5 cm error via this method, but more accurate methods for estimating sea turtle size via UAV (e.g., Piacenza et al. 2022) were not available at the time.

### 2.5 Statistical Analyses

To examine changes in the mean dive duration over time after TurtleCam deployment, we used multilevel modeling to account for any potential effects of animal size. We followed a Bayesian approach to estimate the full distribution of the model's parameters and allow us to quantify support for and against the null hypothesis (Kruschke 2021) of no significant change in dive duration with time after release and no significant differences in dive duration between small or large turtles. Based on the assumption that dive duration would reach a plateau over time, we used a logarithmic model (Equation (1)) with an uncorrelated random intercept and varying slopes between turtle size–classes following a Markov chain Monte Carlo (MCMC) approach for simulations.

(1) $$\text{Dive duration}=\beta_{0}+\beta_{1}∙\ln\left(\text{time}\right)+b∙\ln\left(\text{time}∥\text{Size}\right)$$

where *β*_0_ corresponds to the model's intercept, *β*_1_ to the effect of time, and *b* to the interactions between the intercept and time with size.

After a preliminary analysis, weakly informative priors were determined for intercept ($\beta_{0}\sim N\left(-30,20\right)$) and time ($\beta_{1}\sim N\left(20,10\right)$), respectively. A total of four MCMCs with 3000 iterations each (i.e., 1000 warm‐up and 2000 sampling) were run in the R package “rstanarm” (Goodrich et al. 2020). The full posterior distributions of the model's coefficients were graphically presented in the form of density plots, while 89% credible intervals (CIs) were estimated for each coefficient (preferred to classic 95% intervals for computational stability and better handling of type S error; Gelman and Carlin 2014; Kruschke 2014).

The leave‐one‐out information criterion (LOOIC), which is based on the expected log predicted density (ELPD) of the model and the pareto‐smoothed importance sampling (PSIS) method, was applied to the model (Vehtari, Gelman, and Gabry 2017; Vehtari et al. 2021) to check for the presence of outliers that could affect the fit. The effect of the selected priors on the model's outcome was assessed with a sensitivity test (i.e., refitting with antagonistic priors and comparing). Moreover, an equivalence test of the overlap between the 89% high‐density interval (HDI, in this case identical to the CIs) and the region of practical equivalence (ROPE) quantified the percentage of the credible model solutions that were practically equivalent to the null hypothesis (values other than 0 indicating “nondecisive” evidence; Kruschke 2018). Finally, Bayes factors (BFs; Savage–Dickey density ratio; Wagenmakers et al. 2010) were calculated for each coefficient to quantify the evidence against or in support of the alternative hypothesis *H*_A_ against a complementary alternative *H*_−A_ (i.e., positive effect of each predictor on the outcome vs. negative effect, instead of a null hypothesis *H*_0_ of no effect at all). BF values between 3.2 and 10 constitute “substantial” evidence in favor of *H*_A_ (i.e., positive effect), while values > 10 indicate “strong” evidence (Kass and Raftery 1995). The respective ranges in favor of *H*_−A_ (i.e., negative effect) are 0.3–0.1 and < 0.1. BF values ranging from 0.3 to 3.2 are considered trivial against the null hypothesis *H*_0_ (i.e., no effect). R packages “bayestestR” and “loo” were used for diagnostics and Bayes factors calculations.

## 3 Results

Between September 2018 and March 2020, we successfully deployed and recovered 58 TurtleCams: 31 from Starved Creek, 15 from Rollins Creek, 8 from Deep Creek, 3 from Half Sound, and 1 from Savannah Sound. Of these 58 turtles, 29 were below 50 cm SCL and 29 were above 50 cm SCL (range: 32.6–63.7 cm, mean: 49.7 cm). In total, this resulted in 10,438 min (174.9 h) of TurtleCam footage (range: 122–202.5 min; mean 180 ± 17 min SD).

During the same period, we filmed 25 turtles using UAVs: 4 in Starved Creek, 6 in Rollins Creek, 6 in Deep Creek, and 9 in Half Sound. This resulted in 379 min (6.3 h) of footage (range: 10–20 min, mean: 15 ± 2.93 min SD). Of the turtles filmed via UAV, we estimated that 16 were under 50 cm SCL and 9 were over 50 cm SCL. From this point forward, turtles ≤ 50 cm SCL will be referred to as “small” and those > 50 cm SCL will be referred to as “large.”

A summary of the information on the TurtleCam deployments and UAV surveys is provided in Table S1. TurtleCam deployments and UAV surveys were conducted throughout the year during which the water temperature recorded by the TurtleCams ranged from 21.05°C to 36.2°C. Nevertheless, there was no clear relation between dive duration and mean water temperature except for dives with mean temperature ≤ 22°C, which were slightly longer (Figure S1). Yet as dives with a mean temperature ≤ 22°C constituted only 0.24% of all dives, we did not include water temperature as a key factor in the statistical analyses next.

### 3.1 Postrelease Behavior

**FIGURE 3:**
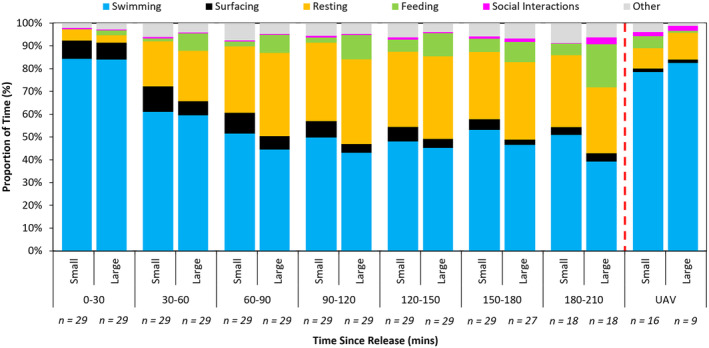
Percentage of time spent by juvenile green turtles of different size–classes (small: ≤ 50 cm SCL, large: > 50 cm SCL) conducting different behaviors (see legend before graph) after the deployment of an TurtleCam. The final pair of columns represent comparable data collected on individuals without biologging devices via unoccupied aerial vehicles. Sample size (*n*) is shown on the *x*‐axis.

For the first 30 min after being released, both small and large turtles still spent 84% of their time swimming (Figure 3), and more time was spent swimming during this time than any other. The next most common behavior during the first 30 min was surfacing at 8% and 7% for small and large turtles, respectively. All the other behaviors were observed for < 5% of the time. The only behaviors that differed by over 1% between small and large turtles were feeding (small: < 1%, large: 2%) and resting (small: 5%, large: 3%).

By 30–60 min, the time spent swimming by both small and large turtles decreased to 61% and 59%, respectively. Simultaneously, the time spent resting and feeding increased over threefold for both small and large turtles. Small turtles spent 20% of their time resting and 1% feeding, while large turtles spent 22% and 8% of the time resting and feeding, respectively. That said, the percentage of time spent feeding by small turtles was still only 1%, while it was 8% for large turtles. From 60 to 90 min, the time spent swimming continued to decrease (small: 52%, large: 44%), while the time spent resting increased (small: 29%, large: 37%).

After 90–120 min and until 180–210 min, the time spent swimming and resting remained relatively consistent for both small and large turtles. Specifically, time spent swimming remained between 48% and 53% for small turtles and between 39% and 48% for large turtles, and for resting it was between 29% and 34% for small turtles and between 29% and 37% for large turtles. In contrast, surfacing, social interactions, and feeding continued to change after 90 min. Specifically, surfacing showed a continual decline until reaching a minimum of 3.7% and 3.4% for large and small turtles respectively. Social interactions increased in large turtles, eventually reaching 3%, though they remained < 1% in small turtles. Finally, feeding increased from 2% to 6% for small turtles and 9% to 19% for large turtles.

Overall, the time utilized in different behaviors was similar for both small and large turtles. The only notable differences were that larger turtles uniformly spent more time feeding, and this continued to increase over time, while it appeared to plateau around 10% for small turtles after 120–150 min. Also, smaller turtles spent slightly more time surfacing and swimming, while larger turtles spent more time resting.

The turtle behaviors identified by UAV were similar for both small and large turtles, with high proportions of time spent swimming (78% and 82% for small and large turtles, respectively). While this was roughly comparable to the proportion of time spent swimming observed between 0 and 30 min after deploying a TurtleCam, turtles recorded by UAV spent far less time surfacing (2% for both small and large turtles) and more time feeding (5% and 2% in small and large turtles, respectively) and in social interactions (1% and 2% in small and large turtles, respectively).

### 3.2 Postrelease Dive Duration

Diagnostic tests confirmed the robustness of the model against both potential outliers (all Pareto's *K* values below the 0.7 threshold) and the initial choice of priors (1.24% deviation for the maximum A posteriori [MAP] probability estimate when fitting with antagonistic priors). Moving to the model's outcome, dive duration increased significantly with time after release (BF for *β*_1_ within the range indicating “strong” evidence in favor of the alternative hypothesis *H*_A_). On the contrary, this increase with time is not affected by turtle size (only “trivial” evidence against the null hypothesis *H*_0_ based on BFs for *β*_3_ and *β*_5_, as well as high overlap of the 89% HDI and the ROPE for *β*_3_ and *β*_5_ based on the equivalence test). Summarized model metrics are presented in Table 2.

**TABLE 2:** Coefficients of the logarithmic model, with standard error, 89% credible intervals (CI), Bayes factors (BF), and the percentage of the high‐density interval in the range of practical equivalence (%HDI in ROPE). Note that CI and HDI values are identical, but the original terminology is maintained. Green and red values mark strong evidence in favor of the alternative hypothesis *H*_A_ and the complementary alternative hypothesis *H*_−A_, respectively.

Model coefficient	Estimate	*SE*	5.5% CI	94.5% CI	BF	% HDI in ROPE
*β*_0_—Intercept	−62.17	39.52	−146.17	17.42	0.09	0.05
*β*_1_—ln(time)	42.32	12.80	2.94	82.03	21.13	0.02
*β*_2_—Intercept:Size “S”	−2.30	23.69	−91.68	63.95	0.77	0.43
*β*_3_—ln(time):Size “S”	3.20	24.01	−59.13	97.20	1.46	0.42
*β*_4_—Intercept:Size “L”	0.77	9.56	−37.81	42.44	1.30	0.68
*β*_5_—ln(time):Size “L”	−0.91	9.37	−43.70	35.34	0.73	0.67

**FIGURE 4:**
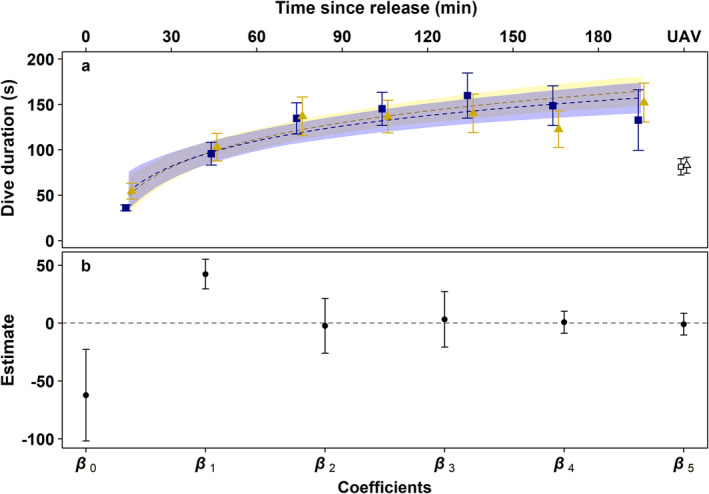
Dive duration of juvenile green turtles of different size classes after the attachment of an TurtleCam or determined via unoccupied aerial vehicles. Upper panel (a): Measured mean dive duration ± SE for small (i.e., ≤ 50 cm; yellow triangles) and large (i.e., > 50 cm; blue squares) turtles during each 30‐min postrelease segment. Hollow symbols at the right side of the panel correspond to the respective measurements with the UAV and do not follow the timeline. Dashed curves indicate the predicted dive durations for continuous time based on the model and are accompanied by 89% posterior intervals (shaded bands). Lower panel (b): Logarithmic model coefficient estimates ± SE (see also Table 2). The dashed line marks 0 (i.e., no effect).

Figure 4a illustrates the measured dive duration of both sets of turtles (i.e., “small” and “large”) at each 30‐min segment after release, alongside overlaid predictions for continuous time based on the means of 1000 posterior draws of the model and, finally, UAV measurements. The most noticeable features are: (i) the overlap of the posterior intervals for both turtle sizes, in accordance with the trivial evidence against *H*_0_ with regard to size (Table 2 rows from *β*_2_ to *β*_5_) and (ii) that measured and estimated dive durations based on TurtleCams at the time of approaching the plateau phase (i.e., “normal” behavior) are notably higher (in fact, double) than the respective UAV values used as “control.” The lower panel (Figure 4b) graphically represents the estimates of the model coefficients (see also Table 2), with *β*_0_ and *β*_1_ ± SE not crossing the 0 line (which would indicate no effect).

The full posterior distributions for all model coefficients, alongside a diagnostic figure of the four MCMC chains, are provided as Figures S2 and S3.

## 4 Discussion

We used animal‐borne cameras and UAVs to quantify how handling stress and biologger attachment impact the short‐term behavior of juvenile green turtles. Our results showed that the portion of time turtles spent swimming was substantially higher in the first 30 min postrelease and did not appear to plateau until 90 min had passed. A high proportion of time spent swimming is unlikely to be a response to animals trying to return to their home range as all turtles were released within 100 m of their encounter location. Instead, it likely reflects a stress‐induced “flight” response that the animal uses to distance itself from the perceived threat. It should be noted that even though we observed a “burst” swimming response (typified by periods of abnormally frantic flipper beats for a few seconds to minutes) in almost all turtles immediately upon returning the animal to the water, this generally only lasted < 1 min (personal observation). In fact, for most of the first 90 min postbiologger deployment, the swimming behavior of the turtles appeared visually “normal” and unstressed. Consequently, it is possible that studies that exclusively consider “burst” swimming as an indicators of disturbance stress in sea turtles (e.g., in response to snorkelers: Griffin et al. 2017, Siegfried et al. 2023 or Bevan et al. 2018) could overlook less conspicuous indicators such as a higher proportion of time spent swimming.

From 90 to 120 min postdeployment onward, the proportion of time turtles spent swimming plateaued at around 40% to 50%. At this time, resting behavior also plateaued around 30%–40% as did mean dive duration with 88.97 ± 89.65 s for small turtles and 108.49 ± 101.72 s for large turtles. Such a plateau could suggest that beyond this point, turtles had resumed “normal” behavior routines that were no longer influenced by handling stress and attachment of the biologger. In support of this assertion, a study on juvenile green turtles in Florida (USA) using triaxial accelerometers to assess activity patterns also reported that they spent around 30% of their time resting (Hart et al. 2016). While our results do show a continued increase in the portion of time that turtles spent foraging after 90 min, this increase was relatively small (< 5%), and by this time the proportion of time that turtles spent foraging in this study was already within the range reported for subadult and adult green turtles in Australia when studied with animal‐borne cameras with a 24‐h delayed start function (Thomson and Heithaus 2014). Thus, it appears that after 90 min behavior of juvenile green turtles has largely returned to “normal” levels.

Our initial goal was to use the UAV data as a “control” to represent the behavior and diving patterns of turtles that had not been handled and did not have biologgers attached. As such, it was assumed that the proportion of time spent conducting the different behaviors and mean dive duration observed with TurtleCams would slowly trend over time toward the values determined from the UAV surveys. Instead, the UAV surveys revealed turtles spent over 80% of their time swimming and relatively short mean dive durations that were comparable to the first 30 min after an animal was released after the attachment of a TurtleCam. In addition, data from the UAV surveys do not appear to fall within the projected ranges of the respective curves as derived in Figure 4. One hypothesis to explain this pattern could be that after the high proportion of time spent swimming in the first 90 min, turtles then take an extended period to rest that exceeds our sampling window (i.e., > 210 min). After a sufficient period of recovery, the turtles return to a higher proportion of time spent swimming again that is comparable to that observed via the UAV surveys. Nevertheless, we consider this hypothesis unlikely considering that other studies that collected data over several days using triaxial accelerometers (Hart et al. 2016) or animal‐borne cameras with delayed start functions (Thomson and Heithaus 2014) show similar behavioral patterns to those that we observed after 90–120 min postdeployment.

A second potential hypothesis that could explain the high proportion of time spent swimming observed in the UAV footage could be that when conducting haphazard UAV surveys to spot turtles, there is bias toward spotting turtles that are actively swimming. Indeed, moving animals are typically much easier to identify than stationary animals, especially in marine systems (Duffy et al. 2018), and the TurtleCams revealed turtles frequently rest under overhanging structures on the seafloor where they may be entirely obscured from the UAV's perspective. A third and final potential hypothesis is that the presence of the UAV at 15 m altitude, while not inducing a “burst” swimming response, may still increase the proportion of time spent swimming in juvenile green turtles. Indeed, noise levels from low‐flying UAVs can reach levels perceivable by marine organisms when near the surface (Christiansen et al. 2016) and there is some evidence that sea turtles will change their behavior in response to low‐frequency noise such as those produced by boat engines (Tyson et al. 2017; Díaz, Kunc, and Houghton 2024). While we think that the second hypothesis is the most probable, both the first and third hypotheses deserve consideration and further study.

We considered reviewing our footage for evidence that turtles were trying to scratch the TurtleCam from their carapace by rubbing against other objects as this could provide evidence that turtles are able to sense the presence of biologgers and can actively try to remove them. While we observed many hours of turtles continually shifting back and forth when resting under substrate, the rhythmic movements of these animals seemed to be passively driven by wave action more than active movements on behalf of the turtle. Turtles are also known to conduct scratching behavior even when no biologging devices have been attached (e.g., Harvey‐Carroll et al. 2024). As such, we have no conclusive evidence that would suggest that turtles actively attempt to remove biologging devices.

### 4.1 Differences in Body Size

There was no visually apparent difference in the mean dive duration between small and large turtles when recorded by the TurtleCam or the UAV (Figure 4). The lack of a difference is interesting considering that body size is often associated with increased lung capacity and longer dive duration in many air‐breathing vertebrates (Mori 2002) including sea turtles (Hochscheid et al. 2007). However, the connection between lung capacity and dive duration assumes that turtles fully inhale their lungs before a dive, and this might not be the case here. Specifically, turtles may limit their lung inhalation volume to reduce their buoyancy when diving (Hays, Metcalfe, and Walne 2004). As buoyancy of the gas in the lungs will decrease with depth in accordance with Boyle's law, turtles with greater quantities of gas in their lungs will need to reach deeper depths to achieve neutral buoyancy. Yet in shallow waters such as those in this study, which never exceeded 5 m depth, turtles may need to only partially inhale to avoid being positively buoyant and thus allow them to rest on the seafloor. This could explain why we did not observe an effect of body size on dive duration and could also explain why the mean dive durations in this study (which after 180–210 min postrelease were still only around 2.5 min), were lower than similarly sized green turtles with access to deeper waters around Australia (Southwood et al. 2003).

With regard to the impact of body size on behavior postbiologger deployment, while there were no substantial differences between small and large turtles, we still observed that smaller turtles always spent more time swimming and surfacing and less time resting and feeding relative to their larger counterparts. Smaller turtles were also first observed feeding between 30 and 60 min, while larger turtles were observed feeding within the first 30 min postdeployment. This suggests that smaller turtles might exhibit a greater stress response to biologger deployment, yet it should be noted that our study only included relatively small juveniles (32.6–63.7 cm SCL), and it would be interesting to determine if these patterns became more pronounced in future studies examining a wider range of body sizes including adult green turtles (70–120 cm SCL).

### 4.2 Methodological Considerations

When attaching the TurtleCams, we used a fast‐setting (< 5 min) epoxy. This allowed us to ensure that handling times with the animal were maintained to within 15–30 min. However, many other studies using epoxy to attach animal‐borne devices onto sea turtles typically use longer setting marine epoxies (see Godley et al. 2008 and references therein) and therefore require animals to be restrained for longer periods of time. It is therefore possible that our results reflect a conservative impact of the typical handling stress on the behavior of juvenile green turtles. That said, as we did not control for handling time in our study, we cannot confirm whether increased handling time also affected the postrelease behavioral response.

To analyze the impact of time and body size on the postrelease diving behavior, we used a Bayesian mixed model with some modifications. First, we opted to use the mean dive duration per turtle per 30 ‐min segment instead of using raw times of each dive. This option reduced our sample size, but it avoided the model providing additional weight to shorter dives, such as those typically performed immediately after release. Second, Bayes factors for time and intercept revealed a significant effect of these two predictors on the dive duration. However, the second metric used (% of HDI in ROPE) did not provide conclusive evidence for that, as there is still a small overlap of the two ranges. This metric is highly conservative, as any overlap between the credible values of a coefficient and the region of practical equivalence (i.e., values that could be equivalent to zero) is interpreted as potentially nullifying significance (Kruschke 2018). In our case, we are confident that this is a matter of differing time scales between the predictor (time) and the outcome (dive duration) variables (i.e., minutes and seconds, respectively). The units chosen are appropriate for the scale of the behaviors being measured, as converting dive durations to minutes or postrelease times to seconds would not provide meaningful comparisons. However, the difference in scale can affect what is considered a “negligible change,” which is essential in defining the ROPE. Thus, given the minimal percentages of overlap, we are confident of the outcome of the model. Finally, the model predicted curves and intervals appear to be higher than the measured dive durations for both turtle sizes after 150 min postrelease. This could be explained by the reduced sample size for these last 30‐min segments (i.e., data for fewer turtles for 150–180 min and 180–210 min), which might have placed more weight on earlier times.

## 5 Conclusions

Our results suggest that the effects of biologger attachment on the behavior and diving duration of juvenile green turtles are largely diminished within 90–120 min postrelease. While we postulate that this is unlikely to have any observable effect on long‐term survival rates, it would still be prudent for further research to consider how the higher proportion of time spent swimming and reduction in feeding behavior influence turtles' daily energetic budgets. Furthermore, we would recommend that studies aiming to generate data on the “natural” behavior and diving patterns of juvenile green turtles consider removing the first 120 min of data postbiologger deployment or program a delayed start for recording data. Finally, the high proportion of time that we observed turtles swimming via the UAV surveys suggests that when using these devices to monitor sea turtle behavior it might be prudent to increase the minimum recommended flying altitude to at least over 15 m. We recommend future studies use animal‐borne cameras with the capacity to film for longer than our 210 min maximum (as have already been achieved in other studies using larger devices, e.g., Jeantet et al. 2020; Hounslow et al. 2023) as well as investigate the capacity for low‐flying UAVs to induce subtle changes in the behavior of sea turtles. We see clear benefits, for both scientific rigor and animal welfare, in refining the use of biologgers and UAVs to minimize any potential for these devices to induce stress or behavioral changes in wild animals.

## Author Contributions

**Nathan J. Robinson:** conceptualization (equal), data curation (equal), formal analysis (equal), funding acquisition (equal), investigation (equal), methodology (equal), project administration (equal), writing – original draft (equal), writing – review and editing (equal). **Ruth Doñate‐Ordóñez:** data curation (equal), formal analysis (equal), writing – review and editing (equal). **Damianos Chatzievangelou:** formal analysis (equal), writing – review and editing (equal). **Annabelle M. L. Brooks:** methodology (equal), writing – review and editing (equal). **Jack Cuffley:** methodology (equal). **Candace Y. A. Fields:** methodology (equal), writing – review and editing (equal). **Sebastian Hoefer:** methodology (equal), writing – review and editing (equal). **Theodora Pinou:** funding acquisition (equal), resources (equal). **Alexander Smith:** methodology (equal), writing – review and editing (equal). **Sophie Mills:** data curation (equal), formal analysis (equal), methodology (equal), writing – review and editing (equal).

## Conflicts of Interest

The authors declare no conflicts of interest.

## Supporting information


Appendix S1.

Appendix S2.

Appendix S3.

## Data Availability

All raw data are uploaded as Appendices S1 and S3.
