# Supplementary material for: Short‐Term Effects of Attaching Animal‐Borne Devices on the Behavior of Juvenile Green Turtles
Source: Ecol Evol. 2024 Dec 23;14(12):e70707. doi: 10.1002/ece3.70707 (PMC11664212; doi:10.1002/ece3.70707)
Supplement: Supplementary file 2 — Appendix S2. [file ECE3-14-e70707-s001.docx]

*SUPPLEMENTARY MATERIALS*

**SHORT-TERM EFFECTS OF ATTACHING ANIMAL-BORNE DEVICES ON THE BEHAVIOR OF JUVENILE GREEN TURTLES**

Nathan J. Robinson^1,2*^, ‬‬‬

^1^Institut de Ciències del Mar, Spanish National Research Council (CSIC), Barcelona, Spain

^2^Fundación Oceanogràfic, Ciudad de las Artes y las Ciencias, Valencia, Spain

^3^University of Algarve, Campus de Gambelas, 8005-139 Faro, Portugal

^4^Cape Eleuthera Institute, Cape Eleuthera Island School, PO Box EL-26029, Rock Sound, The Bahamas

^5^Institute of Environment, Department of Biological Sciences, Florida International University, North Miami, FL, 33181, USA

^6^James Cook University, College of Science and Engineering, Townsville, Australia

^7^Western Connecticut State University, Biology Department, Danbury CT 06810, USA

^8^School of Biological Sciences, Monash University, Victoria, Australia

*Corresponding author: [nathanjackrobinson@gmail.com](mailto:nathanjackrobinson@gmail.com)


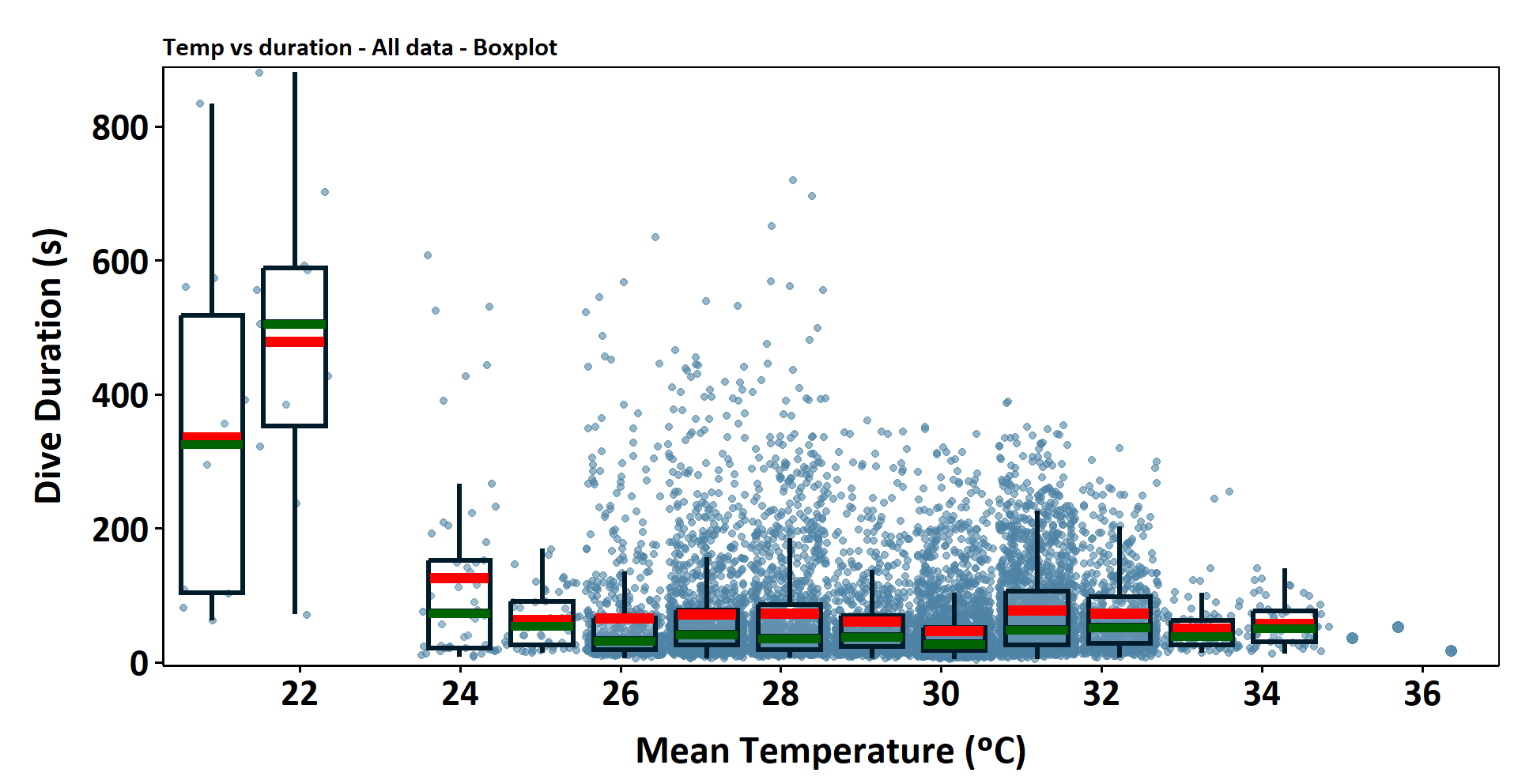


**Figure S1.** Box plots (25 and 75% percentiles) to show dive duration compared to mean water temperature (per 1°C) for all turtles with TurtleCams. The median values are shown as green lines and mean values as red lines. Whiskers are equal to ±1.5 the interquartile range. Light blue dots represent the raw data for each individual diving event.

*Bayesian model diagnostics – MCMC convergence*

We used a total of 4 Markov Chain Monte Carlo chains (MCMCs) with 3,000 iterations each (i.e., 1,000 warm-up and 2,000 sampling), which did not display any prohibitive convergence issues across the six model coefficients (*β_0_* corresponds to the model’s intercept, *β_1_* to the effect of time, and *β_2-5_* to the interactions between the intercept and time with turtle size). All chains are presented in Figure S1.


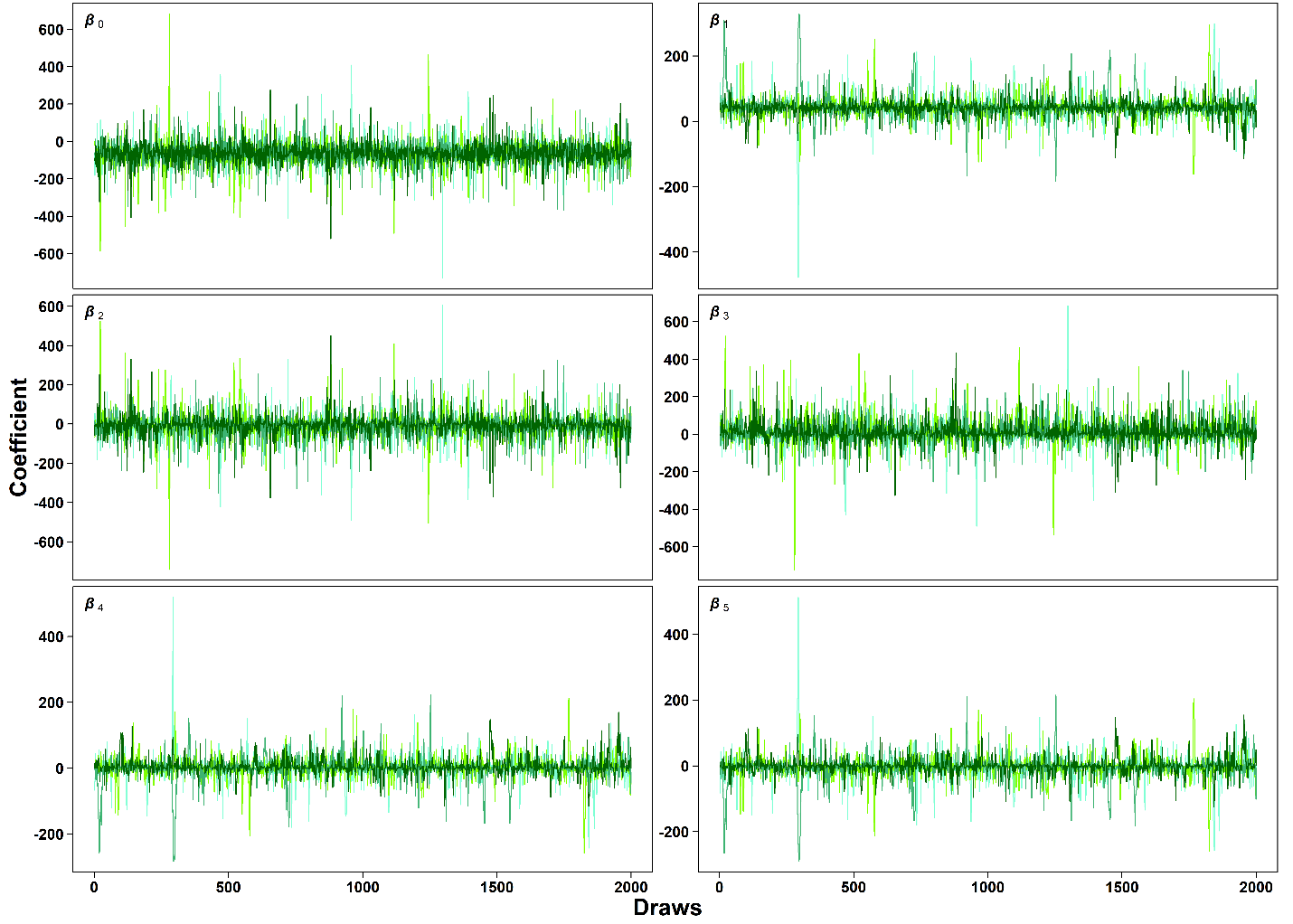


**Figure S2.** MCMC trace plot to assess chain convergence after the 2,000 sampling iterations (previous 1,000 warm-up iterations not shown) for each coefficient of the model. Each MCMC is represented by a different shade of green.

*Bayesian model output – Posterior distributions*

The full posterior distributions (1,000 draws) of the model’s coefficients are displayed in Figure S2, providing high resolution insight on the information presented in Figure 4 of the main text. Regarding the effect of time, 0 falls outside the range of the 89% Credible Intervals, indicating the strongest contribution of this parameter to the model’s variance (Figure S2).


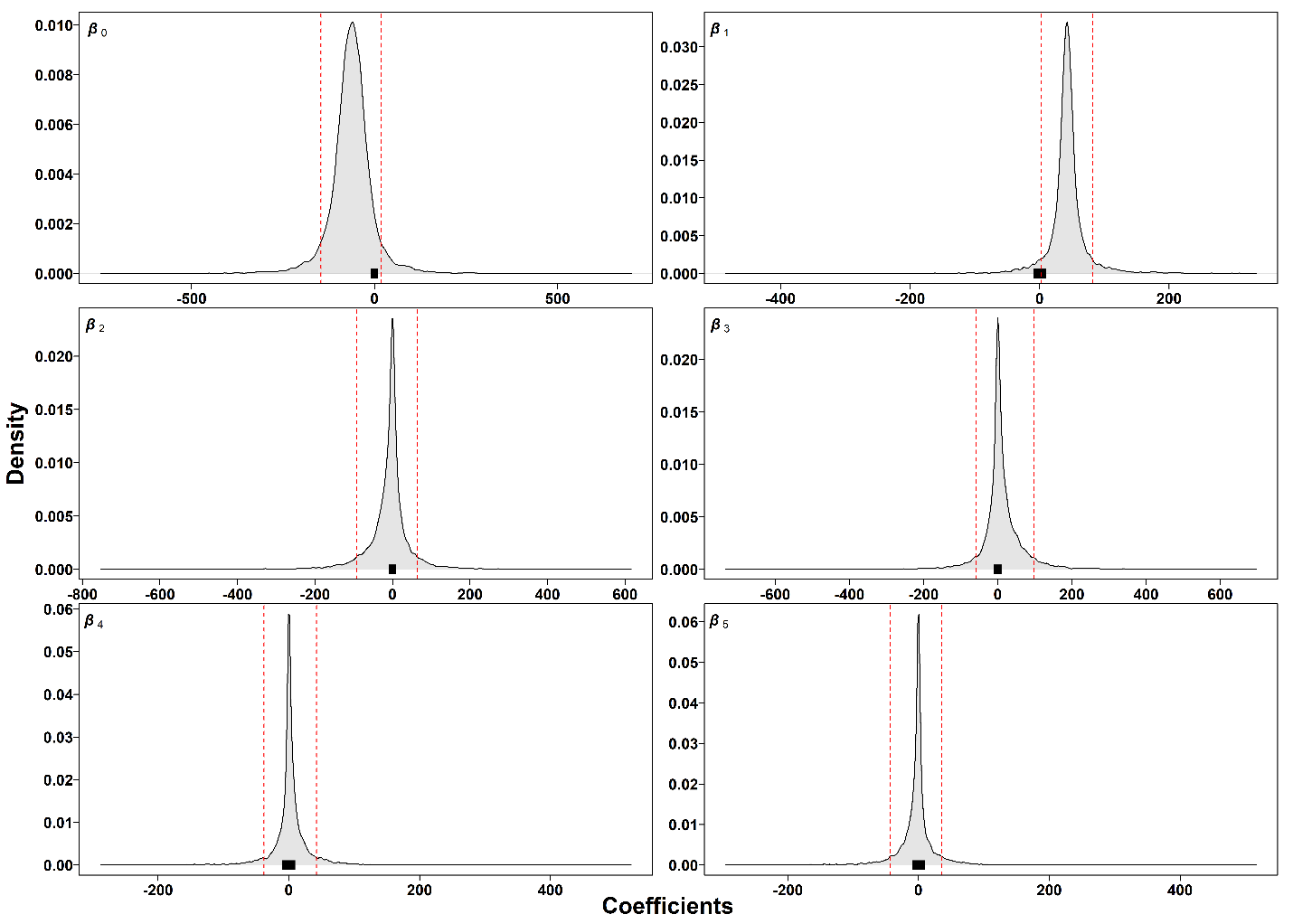


**Figure S3.** Posterior distributions of the model coefficients. Distributions are presented in the form of kernel density plots, where the total area below the curve integrates to 1. The red dashed vertical lines indicate the 89% Credible Intervals. The thick black area around 0 indicates the Region Of Practical Equivalence (ROPE).

**Table S1.** Summary information for TurtleCam deployments and UAVs surveys.

| **Turtle ID** | **Collection method** | **Date** | **Sampling location** | **Body size (cm)** |
| --- | --- | --- | --- | --- |
| TC2 | TurtleCam | 16 – Sep - 2018 | Starved Creek | 53.9 |
| TC3 | TurtleCam | 17 – Sep – 2018 | Starved Creek | 51.7 |
| TC5 | TurtleCam | 20 – Oct – 2018 | Starved Creek | 55.8 |
| TC6 | TurtleCam | 24 – Oct – 2018 | Starved Creek | 51.2 |
| TC7 | TurtleCam | 24 – Oct – 2018 | Starved Creek | 50.6 |
| TC8 | TurtleCam | 31 – Oct - 2018 | Rollins Creek | 40.5 |
| TC9 | TurtleCam | 31 – Oct – 2018 | Rollins Creek | 43.0 |
| TC10 | TurtleCam | 06 – Nov – 2018 | Starved Creek | 59.7 |
| TC11 | TurtleCam | 01 – Oct – 2018 | Rollins Creek | 43.9 |
| TC12 | TurtleCam | 25 – Nov – 2018 | Rollins Creek | 47.7 |
| TC13 | TurtleCam | 25 – Nov – 2018 | Rollins Creek | 48.2 |
| TC14 | TurtleCam | 04 – Dec – 2018 | Starved Creek | 60.9 |
| TC15 | TurtleCam | 14 – Jan – 2019 | Deep Creek | 38.6 |
| TC16 | TurtleCam | 20 – Feb – 2019 | Rollins Creek | 43.1 |
| TC18 | TurtleCam | 01 – Mar – 2019 | Starved Creek | 55.5 |
| TC19 | TurtleCam | 04 – Apr – 2019 | Rollins Creek | 41.4 |
| TC20 | TurtleCam | 04 – Apr – 2019 | Rollins Creek | 44.8 |
| TC21 | TurtleCam | 3 – Oct – 2019 | Deep Creek | 53.4 |
| TC22 | TurtleCam | 17 – Oct – 2019 | Starved Creek | 57.7 |
| TC23 | TurtleCam | 09 – Apr – 2019 | Deep Creek | 46.4 |
| TC24 | TurtleCam | 17 – Apr – 2019 | Rollins Creek | 44.7 |
| TC25 | TurtleCam | 17 – Apr – 2019 | Rollins Creek | 42.6 |
| TC26 | TurtleCam | 25 – Apr – 2019 | Starved Creek | 61.2 |
| TC27 | TurtleCam | 25 – Apr – 2019 | Starved Creek | 53.7 |
| TC28 | TurtleCam | 13 – May – 2019 | Starved Creek | 53.9 |
| TC29 | TurtleCam | 13 – May – 2019 | Starved Creek | 53.4 |
| TC30 | TurtleCam | 04 – Jun – 2019 | Half Sound | 37.2 |
| TC31 | TurtleCam | 04 – Jun – 2019 | Half Sound | 45.6 |
| TC32 | TurtleCam | 05 – Jun – 2019 | Half Sound | 40.3 |
| TC33 | TurtleCam | 01 – Jul – 2019 | Starved Creek | 55.2 |
| TC34 | TurtleCam | 01 – Jul – 2019 | Starved Creek | 32.6 |
| TC35 | TurtleCam | 11 – Jul – 2019 | Starved Creek | 52.9 |
| TC36 | TurtleCam | 11 – Jul – 2019 | Starved Creek | 63.7 |
| TC37 | TurtleCam | 18 – Jul – 2019 | Starved Creek | 55.3 |
| TC38 | TurtleCam | 18 – Jul – 2019 | Starved Creek | 46.2 |
| TC39 | TurtleCam | 21 – Jul – 2019 | Rollins Creek | 43.1 |
| TC40 | TurtleCam | 21 – Jul – 2019 | Rollins Creek | 41.8 |
| TC41 | TurtleCam | 31 – Jul – 2019 | Starved Creek | 57.3 |
| TC42 | TurtleCam | 31 – Jul – 2019 | Starved Creek | 55.8 |
| TC43 | TurtleCam | 7 – Aug – 2019 | Deep Creek | 52.1 |
| TC44 | TurtleCam | 7 – Aug – 2019 | Deep Creek | 47.5 |
| TC45 | TurtleCam | 23 – Aug – 2019 | Rollins Creek | 49.3 |
| TC47 | TurtleCam | 28 – Aug – 2019 | Starved Creek | 47.3 |
| TC48 | TurtleCam | 28 – Aug – 2019 | Starved Creek | 56.8 |
| TC49 | TurtleCam | 05 – Sep – 2019 | Starved Creek | 53.4 |
| TC50 | TurtleCam | 05 – Sep – 2019 | Starved Creek | 47.5 |
| TC52 | TurtleCam | 12 – Sep – 2019 | Starved Creek | 54.1 |
| TC53 | TurtleCam | 26 – Sep – 2019 | Starved Creek | 53.4 |
| TC54 | TurtleCam | 5 – Oct – 2019 | Rollins Creek | 43.7 |
| TC58 | TurtleCam | 22 – Oct – 2019 | Deep Creek | 40.1 |
| TC59 | TurtleCam | 22 – Oct – 2019 | Deep Creek | 44.6 |
| TC60 | TurtleCam | 25 – Oct – 2019 | Starved Creek | 59.2 |
| TC61 | TurtleCam | 25 – Oct – 2019 | Starved Creek | 43.6 |
| TC62 | TurtleCam | 30 – Oct – 2019 | Deep Creek | 55.2 |
| TC63 | TurtleCam | 05 – Dec – 2019 | Starved Creek | 58.3 |
| TC64 | TurtleCam | 19 – Feb – 2020 | Savannah Sound | 55.0 |
| TC65 | TurtleCam | 20 – Feb – 2020 | Starved Creek | 47.5 |
| TC66 | TurtleCam | 12 – Mar – 2020 | Rollins Creek | 46.0 |
| UAV5 | UAV | 15 – Jun – 2019 | Deep Creek | Over 50 |
| UAV6 | UAV | 15 – Oct – 2019 | Half Sound | Under 50 |
| UAV7 | UAV | 15 – Oct – 2019 | Half Sound | Under 50 |
| UAV8 | UAV | 15 – Oct – 2019 | Half Sound | Over 50 |
| UAV10 | UAV | 16 – Oct – 2019 | Deep Creek | Under 50 |
| UAV11 | UAV | 28 – Oct – 2019 | Rollins Creek | Under 50 |
| UAV12 | UAV | 28 – Oct – 2019 | Rollins Creek | Under 50 |
| UAV13 | UAV | 28 – Oct – 2019 | Rollins Creek | Under 50 |
| UAV14 | UAV | 05 – Nov – 2019 | Starved Creek | Under 50 |
| UAV15 | UAV | 08 – Nov – 2019 | Half Sound | Under 50 |
| UAV16 | UAV | 08 – Nov – 2019 | Half Sound | Under 50 |
| UAV17 | UAV | 08 – Nov – 2019 | Half Sound | Under 50 |
| UAV18 | UAV | 10 – Nov – 2019 | Starved Creek | Under 50 |
| UAV19 | UAV | 15 – Nov – 2019 | Half Sound | Under 50 |
| UAV21 | UAV | 15 – Nov – 2019 | Half Sound | Under 50 |
| UAV22 | UAV | 15 – Nov – 2019 | Half Sound | Over 50 |
| UAV23 | UAV | 22 – Nov – 2019 | Rollins Creek | Over 50 |
| UAV24 | UAV | 22 – Nov – 2019 | Rollins Creek | Over 50 |
| UAV25 | UAV | 22 – Nov – 2019 | Rollins Creek | Over 50 |
| UAV26 | UAV | 03 – Dec– 2019 | Starved Creek | Over 50 |
| UAV27 | UAV | 04 – Jan – 2020 | Starved Creek | Over 50 |
| UAV28 | UAV | 28 – Jan – 2020 | Deep Creek | Under 50 |
| UAV29 | UAV | 28 – Jan – 2020 | Deep Creek | Under 50 |
| UAV31 | UAV | 28 – Jan – 2020 | Deep Creek | Under 50 |
| UAV32 | UAV | 31 – Jan – 2020 | Deep Creek | Over 50 |
